# Chronic, low-dose rotenone reproduces Lewy neurites found in early stages of Parkinson's disease, reduces mitochondrial movement and slowly kills differentiated SH-SY5Y neural cells

**DOI:** 10.1186/1750-1326-3-21

**Published:** 2008-12-29

**Authors:** M Kathleen Borland, Patricia A Trimmer, Jeremy D Rubinstein, Paula M Keeney, KP Mohanakumar, Lei Liu, James P Bennett

**Affiliations:** 1 Center for the Study of Neurodegenerative Diseases and Morris K. Udall Parkinson's Disease Research Center of Excellence, University of Virginia, Charlottesville, Virginia, USA; 2 Division of Cell Biology & Physiology, Laboratory of Experimental & Clinical Neuroscience, Indian Institute of Chemical Biology, Calcutta, India; 3 Department of Statistics, Virginia Polytechnic Institute and State University, Blacksburg, Virginia, USA

## Abstract

**Background:**

Parkinson's disease, the most common adult neurodegenerative movement disorder, demonstrates a brain-wide pathology that begins pre-clinically with alpha-synuclein aggregates ("Lewy neurites") in processes of gut enteric and vagal motor neurons. Rostral progression into substantia nigra with death of dopamine neurons produces the motor impairment phenotype that yields a clinical diagnosis. The vast majority of Parkinson's disease occurs sporadically, and current models of sporadic Parkinson's disease (sPD) can utilize directly infused or systemic neurotoxins.

**Results:**

We developed a differentiation protocol for human SH-SY5Y neuroblastoma that yielded non-dividing dopaminergic neural cells with long processes that we then exposed to 50 nM rotenone, a complex I inhibitor used in Parkinson's disease models. After 21 days of rotenone, ~60% of cells died. Their processes retracted and accumulated ASYN-(+) and UB-(+) aggregates that blocked organelle transport. Mitochondrial movement velocities were reduced by 8 days of rotenone and continued to decline over time. No cytoplasmic inclusions resembling Lewy bodies were observed. Gene microarray analyses showed that the majority of genes were under-expressed. qPCR analyses of 11 mtDNA-encoded and 10 nDNA-encoded mitochondrial electron transport chain RNAs' relative expressions revealed small increases in mtDNA-encoded genes and lesser regulation of nDNA-encoded ETC genes.

**Conclusion:**

Subacute rotenone treatment of differentiated SH-SY5Y neuroblastoma cells causes process retraction and partial death over several weeks, slowed mitochondrial movement in processes and appears to reproduce the Lewy neuritic changes of early Parkinson's disease pathology but does not cause Lewy body inclusions. The overall pattern of transcriptional regulation is gene under-expression with minimal regulation of ETC genes in spite of rotenone's being a complex I toxin. This rotenone-SH-SY5Y model in a differentiated human neural cell mimics changes of early Parkinson's disease and may be useful for screening therapeutics for neuroprotection in that disease stage.

## Background

Parkinson's disease (PD) is the most prevalent neurodegenerative movement disorder of adults and occurs sporadically in > 90% of cases. While the primary motor deficits of PD arise from progressive death of dopaminergic substantia nigra neurons, the pathology of PD begins preclinically in lower brainstem nuclei with the appearance in processes of small α-synuclein (+) aggregates ("Lewy neurites"). The Lewy neurite pathology moves rostrally into nigral neurons, followed by death of these neurons, appearance of motor symptoms and formation of larger intracellular aggregates ("Lewy bodies") in surviving nigral neurons [1-6]. In later stages, Lewy neurite pathology appears in limbic and frontal cortex, providing a substrate for the commonly observed neuropsychological clinical problems found in many older PD subjects [7,8].

A comprehensive understanding of PD pathogenesis must account for both the widespread nature of its pathology and the selective vulnerability of nigral dopaminergic neurons. Contemporary and non-exclusionary hypotheses involve the loss of mitochondrial bioenergetic function, impairment of proteasomal activity and appearance of protein aggregates [9-12]. Each of these processes is potentially neurotoxic, and the combination of oxidative stress damage, protein aggregation and altered mitochondrial function has also been described in vulnerable neurons of Alzheimer's brains [13]. Because mitochondrial bioenergetic dysfunction and proteasomal impairment may be interactively linked through oxidative stress, development of complete PD pathology may require dysfunction of both essential cellular components.

To develop an improved cell model for PD pathology, we explored different regimens to differentiate SH-SY5Y dividing neuroblastoma cells into non-dividing differentiated cells that survived for several weeks, formed extensive processes and exhibited a dopaminergic phenotype. We exposed these cells to low concentrations of rotenone to produce ~50% complex I inhibition [14], similar to levels of complex I deficiency reported in PD brain and tissues. We report the effects of such rotenone exposure on neuronal survival, protein aggregation and gene expression, including regulation of mitochondrial electron transport chain genes, and mitochondrial transport along processes.

## Results

### Differentiation and phenotypic analysis

**Figure 1:**
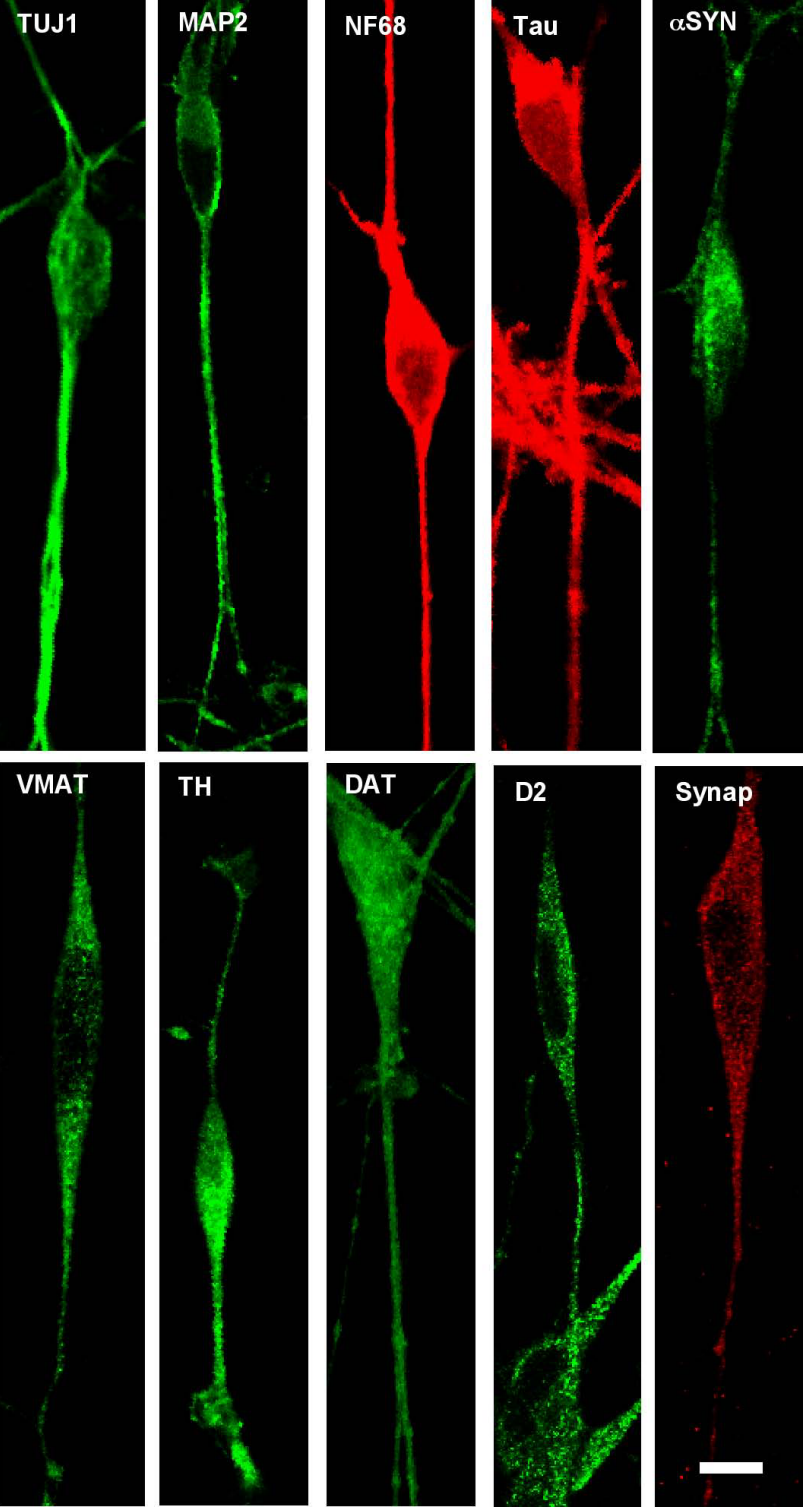
**Confocal microscopic images of immunohistochemical staining of differentiated SH-SY5Y neural cells following differentiation in staurosporine**. Abbreviations: TUJ1 (Neuronal Class III β-Tubulin); MAP-2 (microtubule associated protein 2); NF68 (neurofilament 68); α SYN (α-synuclein); VMAT (vesicular monoamine transporter) TH (tyrosine hydroxylase); DAT (dopamine transporter); D2 (D2 dopamine receptor); Synap (synaptophysin).

The neuronal phenotype of differentiated SH-SY5Y cells was confirmed by labeling with antibodies to the neuron specific beta-tubulin (TUJ1), the 68 kD neurofilament subunit (NF68) and with the microtubule associated proteins MAP-2 and tau (Figure 1). Both MAP-2 and tau were uniformly distributed to the cell body and neurites. Tau was not segregated to the axon-like processes. Synaptophysin and α-synuclein (αSYN) were also detected by immunocytochemistry in differentiated SH-SY5Y cells and exhibited punctuate staining consist with their localization in synaptic vesicles (Figure 1). The dopaminergic phenotype of differentiated SH-SY5Y cells was confirmed by immunostaining with tyrosine hydroxylase (TH), dopamine transporter (DAT), the vesicular monoamine transporter (VMAT) and the D2 dopamine receptor (D2) (Figure 1). The dopaminergic markers also exhibited punctuate staining in the cell body and processes of SH-SY5Y cells in culture. As part of a separate study we have also confirmed that staurosporine-differentiated SH-SY5Y cells express choline acetyltransferase (data not shown), indicating that these cells continue to express multiple neurotransmitter types.

### Rotenone-induced formation of Lewy neurite-like structure

**Figure 2:**
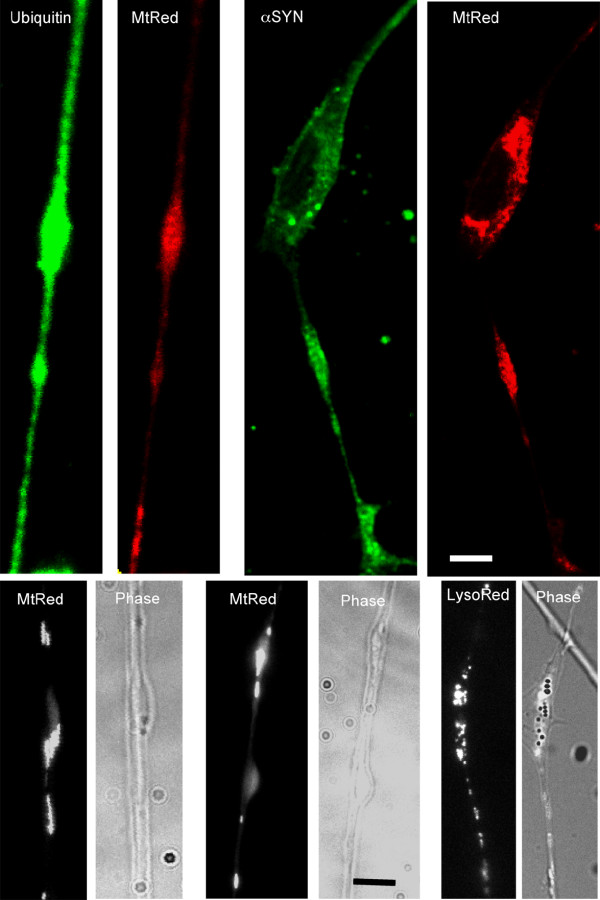
**Lewy neurites in processes of differentiated SH-SY5Y neural cells after incubation with 50 nM rotenone for 7 days**. MtRed = MitoTracker Red; α SYN = alpha synuclein; Phase = phase contrast microscopy; LysoRed = LysoTracker Red.

**Figure 3:**
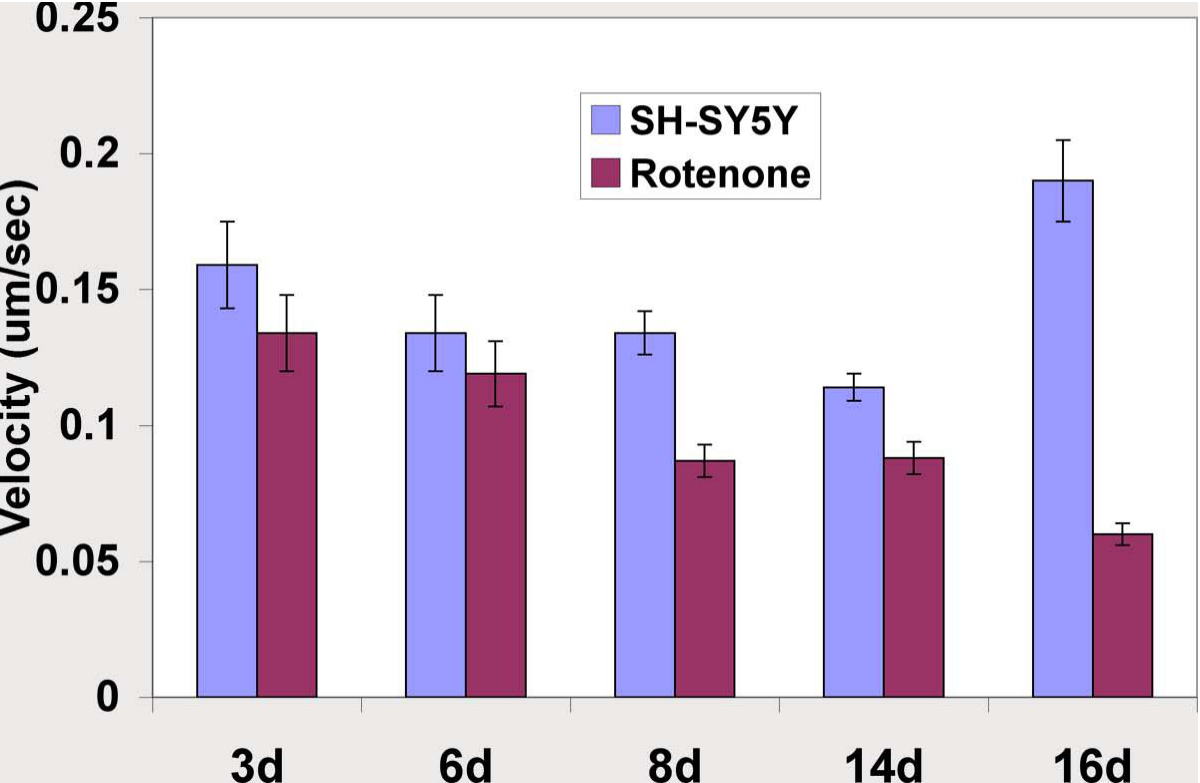
**Effects of 50 nM rotenone exposure on mitochondrial movement velocities in processes of differentiated SH-SY5Y neural cells**. The X-axis shows duration of rotenone treatment; Y-axis shows mean +/- SEM mitochondrial movement velocities (microns/sec).

Chronic treatment with 50 nM rotenone induced the formation of neuritic swellings beginning at day 3 and continuing for up to 20 days. The number of neuritic swellings per culture was low but they were readily identifiable. Cultures of staurosporine-differentiated, rotenone-treated SY5Y neural cells were stained with antibodies to α-synuclein and ubiquitin (Figure 2). These neuritic swellings morphologically resemble the α-synuclein and ubiquitin immunoreactive Lewy neurites seen in increasing numbers in PD brain samples [1-4]. Neuritic swellings generated by rotenone exposure also contained concentrations of bioenergetically competent mitochondria, based on their capacity to accumulate the potentiometric dye MitoTracker CMX-Ros (MtRed) and LysoTracker positive lysosomes (Figure 2). The dynamic movement of mitochondria in rotenone-treated neurites exhibiting neuritic swellings was reduced in comparison with intact neurites (Figure 3). Reduction in mitochondrial movement was statistically significant by 8 days of rotenone treatment and was greatest at 16 days, the longest period studied.

### Rotenone-induced cell death

**Figure 4:**
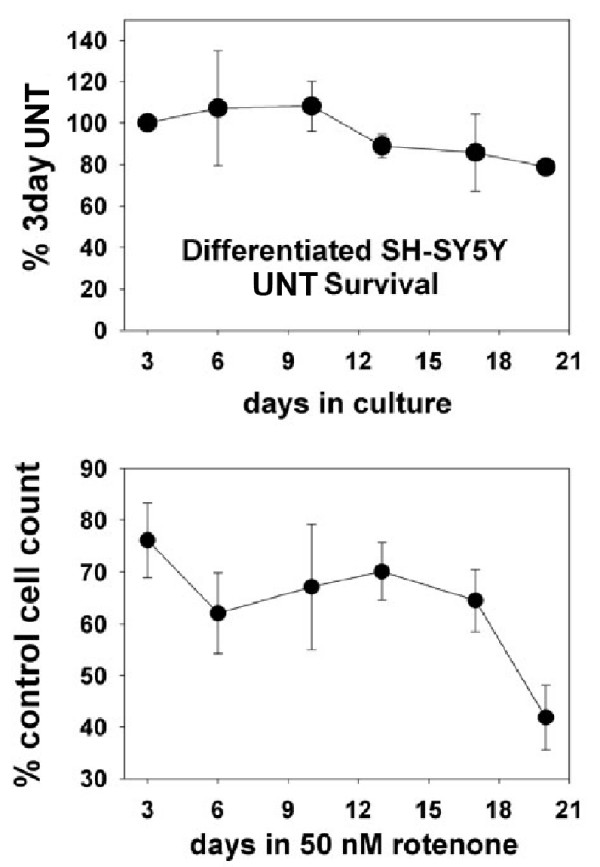
**Survival of differentiated SH-SY5Y neural cells during exposure to 50 nM rotenone**. Shown are the means +/- SEM's of three independent experiments in which population densities of SH-SY5Y cells were determined from counting calcein (+) cells over the course of exposure to 50 nM rotenone.

**Figure 5:**
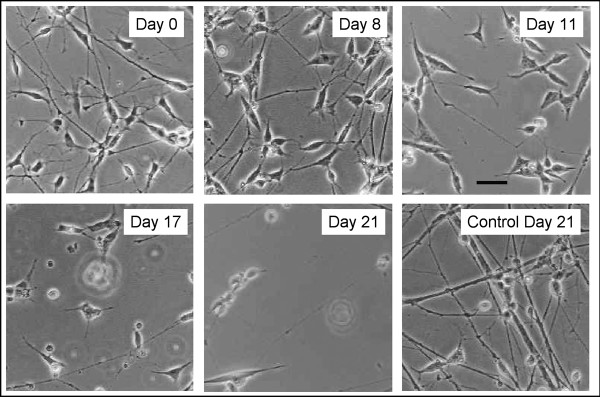
**Phase contrast images of differentiated SH-SY5Y cells chronically exposed to 50 nM rotenone**.

**Figure 6:**
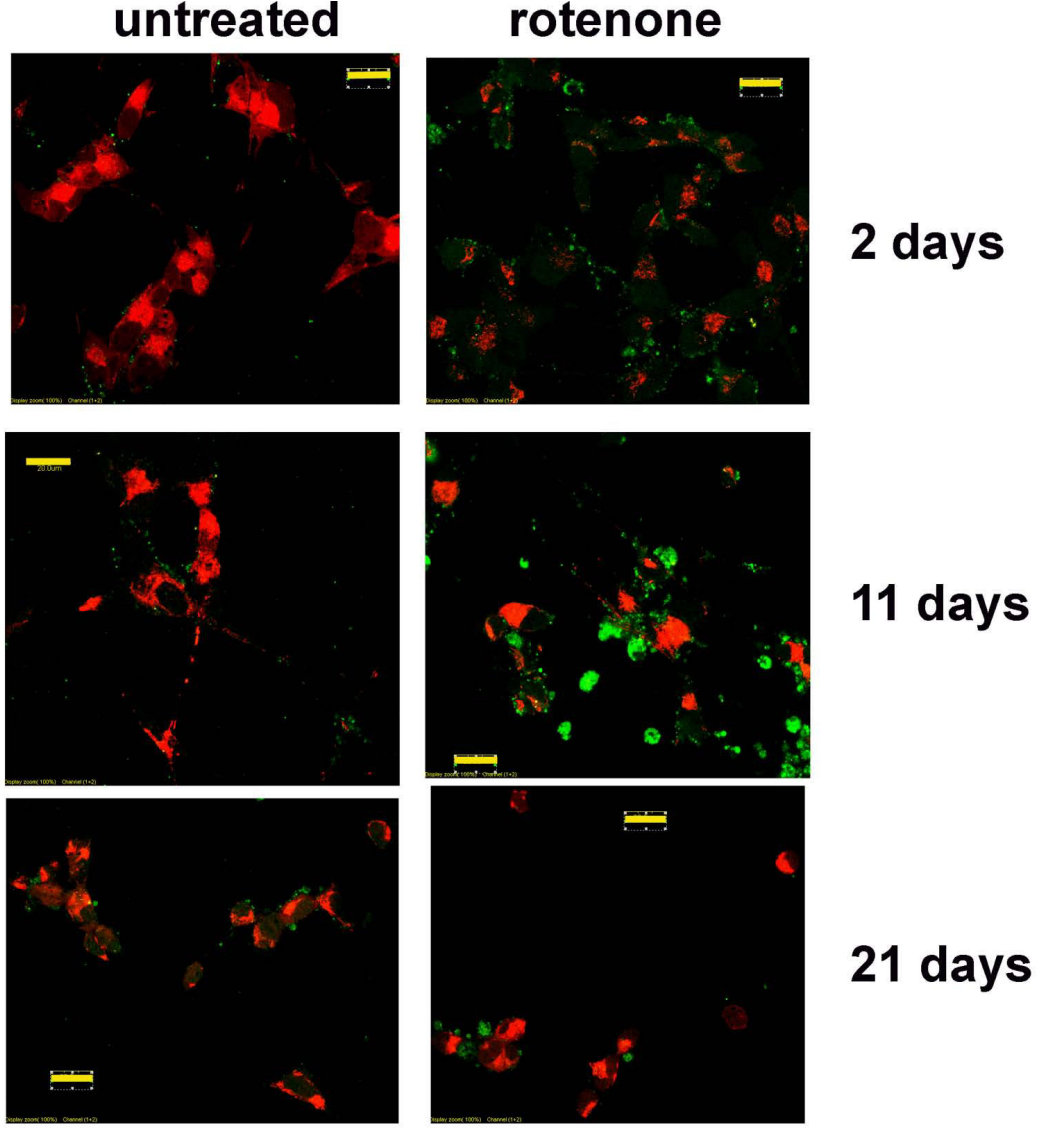
**Confocal microscopic images of differentiated SH-SY5Y neural cells exposed to 50 nM rotenone for the indicated number of days after staining with zVAD.fmk-FITC (green) to indicate activated caspases**. Mitochondria labeled with MitoTracker Red.

**Figure 7:**
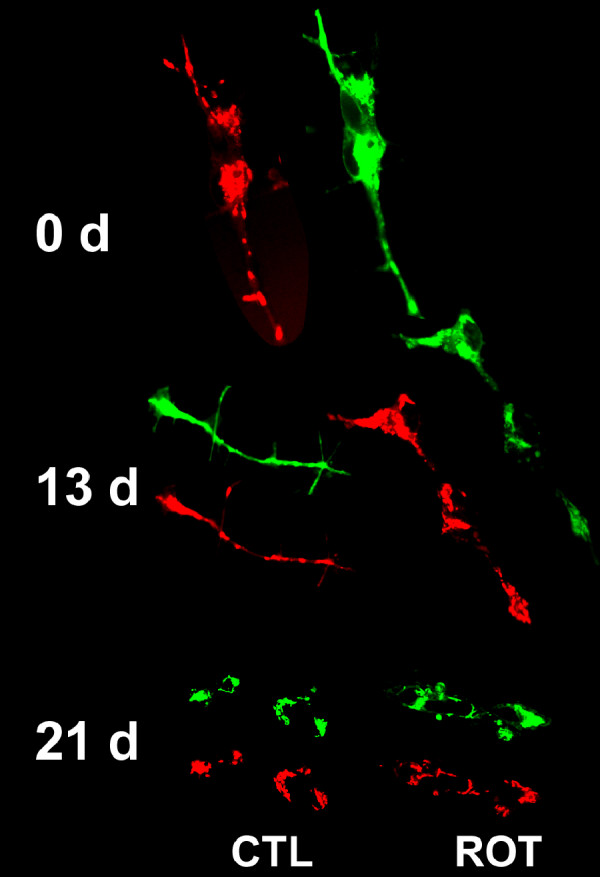
**Images of JC-1 staining of differentiated SH-SY5Y neural cells exposed to 50 nM rotenone for the indicated number of days**. Green corresponds to monomeric JC-1 accumulating in low voltage mitochondria, and red corresponds to J-aggregates that form in highly energized mitochondria. Shown are representative examples of green/red image pairs at different rotenone exposure times. Cells with loss of red JC-1 aggregate staining were not observed at any rotenone exposure time point. Note the absence of neurites in the 21 days neurons.

50 nM rotenone produced a biphasic survival curve with ~40% loss by 6 days and a second decline to ~60% loss between 18 and 21 days (Figure 4). The differentiated cells surviving in rotenone exhibited progressive loss of processes (Figure 5). General activation of caspases, monitored with zVAD.fmk-FITC staining, was visible by 2 days of rotenone at a low level with small intense foci (Figure 6). By 11 days of rotenone more generalized caspase activation was noted that was still visible at 21 days (Figure 6). We did not observe any evidence for loss of mitochondrial membrane potential, monitored with JC-1 staining (Figure 7).

### Gene Expression Changes

#### Microarray results

**Figure 8:**
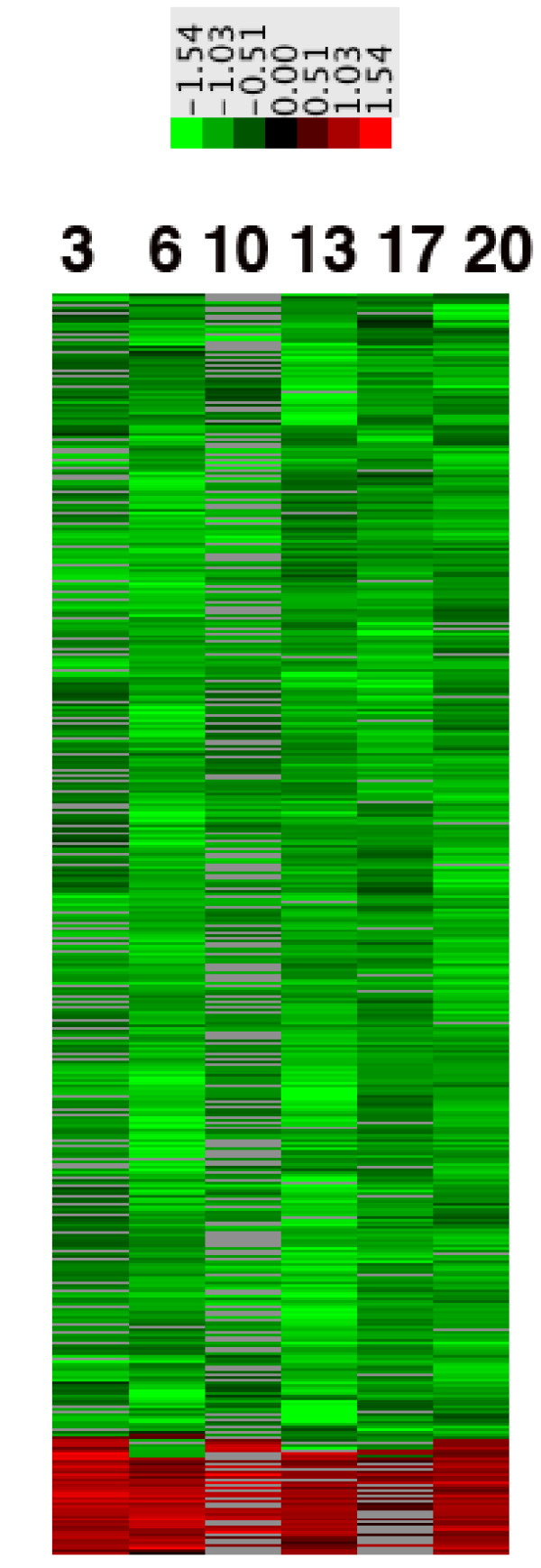
**CLUSTER heatmap showing distributions of expression ratios for named genes from differentiated, rotenone-treated SH-SY5Y neural cells**. Total RNA samples from the three independent rotenone incubation experiments were hybridized in duplicate on 19.2K glass microarrays. Following analysis for significance with B.A.G.E.L. software, the transcripts with P < 0.05 significance were annotated with DAVID to named genes and processed with CLUSTER to generate the heatmap. Shown are the distributions of the 1200 genes that were present in at least 50% of the samples across the time (days) of rotenone exposure. Gray spaces indicate that a particular transcript was not present. The colorbar above the heatmap shows the natural logarithm of the averaged rotenone/CTL values.

Figure 8 shows a Cluster heatmap for distribution of expression of named genes across time (days) of rotenone exposure. The Cluster analysis was performed so that at least 50% of the samples contained a value. This narrowed the initial gene list of 10,774 genes present on the array to 1200. The data shown represent the averaged ROT/CTL ratios from three independent, sequential experiments for transcripts that reached at least P < 0.05 by the B.A.G.E.L. algorithm. None of these populations was corrected for multiple comparisons.

The rotenone-treated, differentiated SH-SY5Y cells had similar overall patterns of generally reduced expression, and there were cyclical increases in densities of under-expressed transcripts at 6d, 13d and 20d of rotenone exposure. A minority of genes were over-expressed and showed increased densities at 3d, 6d, 13d and 20d of rotenone exposure.

#### qPCR analysis of relative expression of mitochondrial ETC genes

**Figure 9:**
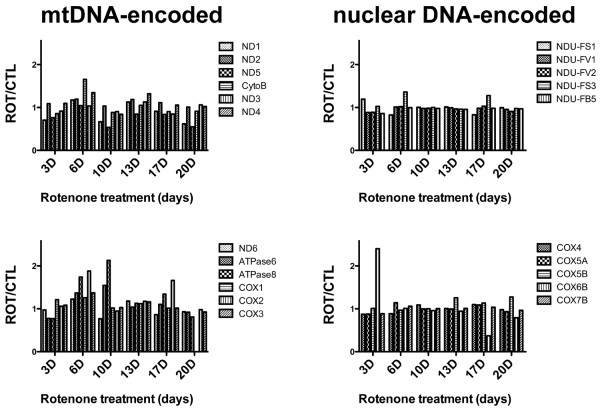
**Graphs showing changes in ROT/CTL ratios across duration of ROT exposure (days) for mtDNA-encoded (left) and nuclear DNA-encoded (right) ETC gene expressions assayed in cDNA samples by qPCR**. Shown are averages from three independent experiments.

We also determined relative expression levels for mitochondrial ETC genes by using qPCR to assay levels of 12 mtDNA-encoded (Figure 9, left) and 10 nDNA-encoded (Figure 9, right) ETC genes in total RNA's from all three sets of rotenone-treated and non-treated, differentiated SH-SY5Y cells. Across the duration of rotenone exposure there were no substantial changes in expression of mtDNA-encoded complex I genes, with increases at 6d and 10d for both ATP synthase genes and an increase for CO subunit 2 at 6d. The maximum detectable increases in relative expression for mtDNA-encoded genes ranged between 1.5–2.2. There were minimal changes in regulation of nDNA-encoded ETC genes.

## Discussion

The major conclusion from our study is that it is possible to create a rotenone-based model of human neuron degeneration that recapitulates aspects of protein aggregation and neuritic pathology of early sPD. In our model, differentiated, non-dividing SH-SY5Y dopaminergic neural cells experienced gradual death over 3 weeks of exposure to a [rotenone] that produces ~50% inhibition of complex I activity, comparable to that found in PD brain and peripheral tissues. Based on preservation of ΔΨ_M _in surviving cells, we found no evidence of bioenergetic deficiency in terms of potential to synthesize ATP. Although heterogeneous caspase activation was observed, our methodology does not define which caspase(s) are activated, and it is presently unknown how this activation occurred or whether it is causal for the neuronal death that takes place.

The accumulation of functional mitochondria and lysosomes in neuritic swellings suggests that axonal transport is impaired at these sites. Our studies of mitochondrial movement in differentiated, rotenone-treated SH-SY5Y neural cells with neuritic swellings showed that the transport of mitochondria was progressively reduced over the course of rotenone exposure. Several different mechanisms may contribute to reduced transport in processes containing neuritic swellings. In addition to binding to complex I, rotenone also binds to microtubules and disrupts vesicular transport [15]. While this would explain a total reduction in axonal transport in rotenone-treated neurons, it would not explain the formation of neuritic swellings. Rotenone treatment induces oxidative stress, which has been shown to contribute to axonal swelling and beading in cultured neurons [15,16]. These authors suggest that free radical damage can cause focal disruption of mitotubules and induce the formation of axonal swellings.

Rotenone exposure also elevates the expression of soluble and insoluble α-synuclein in human neuroblastoma cells in culture [17]. Over-expression of α-synuclein is detrimental to the microtubule system and results in neuritic degeneration, trafficking defects and Golgi fragmentation [18]. The formation of α-synuclein aggregates may also contribute to the generation of neuritic swellings in differentiated, rotenone-treated SH-SY5Y neural cells. In a similar vein, huntingtin aggregates can impair mitochondrial trafficking in cortical neurons [19]. In this study the aggregates blocked the passage of mitochondria in processes causing them to accumulate adjacent to the aggregates and become immobile.

The presence of α-synuclein in rotenone-induced neurite swellings suggests that aggregates of a-synuclein may form in neurites and impede the movement of mitochondria and lysosomes. Unlike PD cybrids generated from SH-SY5Y neuroblastoma cells [20], rotenone-treated, differentiated SH-SY5Y neural cells did not generate cybrid Lewy bodies. This suggests that rotenone treatment induces only certain early aspects of PD related pathological changes that involve protein aggregation.

It is noteworthy that the effects of rotenone exposure on transcript changes were qualitatively similar across time with an appearance of cyclical increases in under-expression and over-expression. These findings suggest that at the transcriptional regulation level neuronal cell death is a temporally dynamic process with "waves" of gene expression changes. However, in our model system the amplitudes of these changes in gene expression across rotenone exposure were small and certainly not defining of a major effect.

A few mitochondrial ETC genes coded for by mtDNA, but none of the mtDNA-encoded complex I genes, showed increased expression at 6 and 10 days of rotenone exposure, but ETC genes coded by nuclear DNA, including several complex I genes, generally changed very little. Because rotenone is a complex I toxin, this was a surprising finding and implies little cellular response to partially impaired mitochondrial function. Overall, our findings with gene expression changes suggest that in this model system they are not likely to provide robust biomarkers for screening therapeutics.

One important caveat to this study is that although the SH-SY5Y cells were differentiated to become non-dividing cells with a neuronal phenotype, they are still derived from a neuroblastoma tumor lineage and are not primary neurons. This neoplastic lineage may influence gene expression responses to subacute rotenone exposure. We considered using rodent midbrain primary neurons but decided to stay with the differentiated SH-SY5Y for three reasons. First, they are human cells, and we felt it was problematic to compare gene expression responses across species-specific arrays. Second, they are both the host cells for our PD cybrids and the cells used in other gene expression studies, and we wished to be able to compare expression datasets. Finally, we wanted to develop a rotenone-based cell culture model for PD with human neural cells, and the SH-SY5Y line seemed ideal for that purpose. As differentiated human stem cell-derived dopaminergic neurons become more widely available, it will be important to substitute them for this type of study.

## Conclusion

Subacute, low-dose rotenone treatment of differentiated SH-SY5Y neural cells produced heterogenous caspase activation, neuritic protein aggregation pathology and process retraction that could be considered as similar to that occurring in early PD. Because we did not have access to PD brain tissue (esp substantia nigra) from "early" disease cases, it is not possible for us to compare our gene expression changes to those of PD nigral neurons at the stage where neuritic pathology is first appearing. Such a comparison would be valuable to determine if our differentiated, rotenone-treated SH-SY5Y neural cell model recapitulates early gene expression changes of PD nigral neurons, instead of those found with typical autopsy material that is from persons with late stage disease. With that limitation in mind, we suggest that our differentiated SH-SY5Y neural cells exposed to low [rotenone] are a viable model for pathological changes of early PD and could be used for screening neuroprotective drugs, particularly those that might prevent the initial pathologies of process retraction and Lewy neurite formation. Based on our results, we do not feel that gene expression changes in this model are likely to be helpful in therapy development, as the changes we found were of too low a magnitude to provide a meaningful signal. However, improvement in impaired mitochondrial transport may represent a meaningful biological signal to predict reduction of process retraction and cell death.

## Methods

### General

SH-SY5Y cells were routinely cultured in growth medium (GM), consisting of high glucose DMEM with 10% FBS, 100 μg/ml pyruvate, 50 μg/ml uridine, and antibiotic-antimycotic: 100 Units/ml penicillin G, 100μg/ml streptomycin, 0.25 μg/ml amphotericin β. Cells were fed every 2–3 days and passed once a week. Cells were differentiated by culturing in Neurobasal medium and adding staurosporine at 6–10 nM (differentiation medium, DM).

### Rotenone treatment

At the end of differentiation, samples were collected for Day 0 data and medium was changed to begin 50 nM rotenone treatment on selected flasks and dishes. Both rotenone and staurosporine were added, fresh, to DM at each medium change.

### Immunocytochemistry and Confocal Microscopy

To immunostain differentiated SH-SY5Y neural cells, cells were grown in 35 mm glass bottom culture dishes [21], fixed with 4% paraformaldehyde in 0.1 M phosphate-buffered saline (PBS), rinsed with PBS and incubated in blocking solution (0.05% bovine serum albumin and 0.2% triton X-100 in PBS) for 30 min at room temperature. Then primary antibodies for each antigen were diluted in PBS containing a 1:10 dilution of blocking buffer and incubated overnight at 4°C. The primary antibodies used for this study include-α-synuclein (1:500, AB5038), dopamine transporter (1:1000, MAB369), neurofilament 68 (1:100, AB1983), synaptophysin (1:100, MAB368), tau (1:100, AB1512), tyrosine hydroxylase (1:1000, AB151), ubiquitin (1:250, MAB1510) and vesicular monoamine transporter (1:200, AB1767) from Chemicon International, Inc. (now owned by Millipore, Temecula, CA). The antibody for the D2DR dopamine receptor (1:100, sc-9113) was obtained from Santa Cruz Technology Inc. (Santa Cruz, CA), the microtubule associated protein 2 was purchased from (MAP-2, 1:62, 13–1500) Zymed now owned by Invitrogen, (Carlsbad, CA), and the Neuronal Class III ^®^-Tubulin was obtained from Dr. Anthony Frankfurter (University of Virginia). After 3 washes in PBS, the cells were incubated in appropriate fluorescein or Texas-red labeled secondary antibodies (1:100, Vector Labs, Burlingame, CA) for 30 min at room temperature and mounted with Vectashield (Vector Labs, Burlingame, CA). The fluorescently-labeled anti-rat secondary antibodies needed to image the dopamine transporter primary antibody were purchased from Jackson ImmunoResearch Labs, Inc (West Grove, PA). Images of stained cybrid neurons were created using an Olympus Fluoview laser scanning confocal microscope system.

To label mitochondria, differentiated cybrid cells were incubated with 15 nM MitoTracker CMXRos (Invitrogen, Carlsbad, CA) for 10 min at 37°C. After a brief wash in GM, the cells were imaged using an Olympus IX70 inverted microscope equipped with epifluorescence and Nomarski optics, a Lambda 10-2 filter wheel, a Photometrics CoolSnap HQ progressive scan CCD camera and MetaMorph software (Molecular Devices). Mitochondrial movement was visualized and quantitated as described [22]

### Confocal microscopic imaging of activated caspases using FITC-zVAD.fmk

Differentiated SH-SY5Y cells were grown and treated with rotenone and staurosporine in 35 mm dishes as described above. Labeling solutions and labeled cells were protected from light during the following steps. To label suspended cells, 40–50 ml of "spent" medium was collected from 20 dishes, centrifuged at 250 × g for 8 min, the pellet was resuspended in 400 ul Dulbecco's Modified Eagle Medium with high glucose, HEPES buffer and without phenol red (Gibco-Invitrogen, Grand Island, NY) and 200 ul plated on the coverslip area of two, 1% porcine skin gelatin-coated, 35 mm dishes. After 30 min at 37°C, 100 ul of HEPES medium containing 30 uM CaspACE FITC-zVAD-FMK (Promega, Madison, WI) and 240 nM MitoTracker Red (Molecular Probes, Eugene, OR) was added and the cells incubated for another 30 min at 37°C. To label attached cells, maintenance media was removed and cells incubated for 30 min. at 37°C in HEPES medium containing 10 uM CaspACE FITC-z VAD-FMK and 80 nM MitoTracker Red. All labeled dishes were washed with Hank's Balanced Salt Solution (Gibco-Invitrogen, Grand Island, NY), fixed for 20 min in 4% phosphate buffered paraformaldehyde with 4% sucrose, pH 7.4, washed with PBS and coversliped using Vectashield mounting medium (Vector Laboratories, Burlingame, CA). Single plane confocal images of labeled cells were collected using an Olympus Confocal Laser Scanning System mounted on an Olympus IX70 microscope.

### Microarray studies

We examined gene expression in differentiated SH-SY5Y after 3, 6, 10, 13, 17 and 20 days of exposure to 50 nM rotenone. Our approach to microarray studies was to emphasize both technical and biological replication. To achieve those goals, we carried out three temporally consecutive, independent studies of rotenone exposure. SH-SY5Y were differentiated as above and then carried forward in culture with or without 50 nM rotenone. At each time point of rotenone exposure, RNA was harvested from rotenone-exposed and non-exposed companion cultures. This procedure attempted to control for any effects of the differentiation procedure and "aging" in culture, leaving rotenone exposure as the only variable.

RNA samples were reverse transcribed to fluorescein labeled (CTL cells) or biotin labeled (ROT cells) cDNA using the Micromax TSA™ Labeling and Detection Kit (Perkin Elmer Life and Analytical Sciences, Inc., Waltham, MA). ROT samples for each time point were each assayed against the corresponding CTL SH-SY5Y cells.

The two labeled cDNAs were then mixed and hybridized to a 19.2K human gene array (University Health Network Microarray Center, Toronto, Ontario) overnight at 40°C in a humidified chamber. Detection and amplification of signal were accomplished using a series of antibody-enzyme conjugates catalyzing the deposition of Cyanine 3 tyramide (Cy3) to CNT or UNT cDNA and Cyanine 5 tyramide (Cy5) to PD or ROT cDNA. Arrays were hybridized in duplicate and imaged in a scanning confocal slide reader Scanarray^® ^4000, (Packard Biochip Technologies, LLC, Billerica, MA). Random dye-swap experiments with arrays and labeling reagents from the above manufacturers always produced > 90% concordance for expression levels.

### Statistical processing and gene ontology analysis

Duplicates of background-subtracted, normalized to total array fluorescence transcript fluorescence ratios (Rot/Ctl) were first averaged then processed for significance using B.A.G.E.L. (Bayesian Analysis of Gene Expression Levels, [23]). The B.A.G.E.L. algorithm makes no assumptions about what expression levels are "biologically significant" and instead calculates significance for a given transcript based on its variance compared to the overall population variance. In the B.A.G.E.L. analyses performed, all three independent rotenone experiments at each time point of rotenone exposure were analyzed together.

We used the B.A.G.E.L. statistical output to devise a liberal approach to filter transcripts used for subsequent analysis. To analyze transcript expression patterns, we first collected all transcripts at each rotenone treatment time point or in the PD brain samples that achieved p < 0.05 significance in the B.A.G.E.L. analysis and were not corrected for multiple comparisons. The GenBank transcripts were then annotated to gene symbols using D.A.V.I.D. (Database for Annotation, Visualization and Integrated Discovery, ) and analyzed using the Cluster [24] algorithm to create visual patterns of gene changes across time of rotenone exposure compared to those observed in the PD brain samples. We utilized the latest version of Cluster 3.0 to perform hierarchical clustering and JavaTreeView (v. 1.0.12, ) to view the Cluster output.

### Real-time, quantitative PCR (RT-qPCR)

Primers for mtDNA and nDNA-encoded ETC genes were designed with Beacon Designer software and used with SyberGreen detection to assay relative gene expression in cDNA's prepared by reverse transcription of the RNA samples. All assays were carried out in an iQ5 instrument (BioRad). Relative gene expression was calculated with the method of Pfaffl [25].

## Competing interests

The authors declare that they have no competing interests.

## Authors' contributions

MKB developed the differentiation protocol, performed the rotenone exposure experiments, extracted RNA and performed the microarray studies; PAT designed experiments, carried out the mitochondrial movement studies and wrote the manuscript; JDR and KPM performed the qPCR studies; PMK did the confocal microscopy experiments; LL carried out statistical analyses; JPB designed experiments, analyzed microarray and qPCR data and wrote the manuscript.
